# Clinical Management of Pneumonitis in Patients Receiving Anti–PD-1/PD-L1 Therapy

**Published:** 2018-05-01

**Authors:** Justin E. Bala-Hampton, Angela F. Bazzell, Joyce E. Dains

**Affiliations:** The University of Texas MD Anderson Cancer Center, Houston, Texas

## Abstract

**CASE STUDY**

A 48-year-old gentleman with metastatic melanoma currently receiving the cytotoxic T-lymphocyte–associated antigen 4 (CTLA-4) inhibitor, ipilimumab (Yervoy), and the programmed cell death protein 1 (PD-1) inhibitor, nivolumab (Opdivo), returned for evaluation prior to receiving cycle 2. The patient presented with new onset dyspnea and a non-productive cough over the past week, with a temperature of 100.6°F at home on one occasion. He was placed on observation for fever, cough, and shortness of breath. The patient had no previous history of lung disease and was a nonsmoker. Diminished breath sounds were noted on auscultation. However, the patient was without fever or chills, with a heart rate of 101 beats per minute and a blood pressure of 110/75 mm Hg.

We obtained a computed tomography (CT) of his chest. The CT demonstrated diffuse ground-glass opacities in his bilateral lower lobes and some minor interstitial thickening of his right middle lobe, possibly suggestive of inflammation or cryptogenic organizing pneumonia.

Based on his presentation and CT findings, the patient was initially treated empirically with antibiotics for suspected pneumonia vs. pneumonitis. During the first 12 hours in observation, the patient experienced increasing dyspnea and cough and was admitted to the hospital. Nebulizer treatments were administered with no improvement, so the patient was started on high-dose corticosteroids at 1 mg/kg, and pulmonary and infectious disease consults were ordered. After the administration of steroids, the patient’s cough and breathing improved and he remained afebrile, eliciting a high suspicion for immune-related pneumonitis. The patient then underwent bronchoscopy to rule out other etiologies.

Bronchoalveolar lavage was performed, which yielded no pathogenic organisms. The patient was placed on a 3-week course of a high-dose steroid taper, following which immunotherapy was reinstated. Within 4 days he again presented with similar symptoms, was restarted on high-dose steroids, and immunotherapy was permanently discontinued.

Immune checkpoint blockade monoclonal antibodies have revolutionized treatment and improved the prognosis for many patients with cancer (Eigentler et al., 2016; Weber, Yang, Atkins, & Disis, 2015; Wu, Hong, Zhang, Lu, & Miao, 2017). Immunotherapeutic drugs approved by the US Food and Drug Administration (FDA) since 2011 include inhibitors of cytotoxic T-lymphocyte–associated antigen 4 (CTLA-4), programmed cell death protein 1 (PD-1), and its ligand (PD-L1; Buchbinder & Desai, 2016; Medina & Adams, 2016). These novel treatments utilize various checkpoint pathways to interfere with antitumor immune responses, resulting in increased activation of the body’s immune system (Buchbinder & Desai, 2016).

Currently, anti–PD-1/PD-L1 checkpoint antibodies are approved by the FDA to treat melanoma, non–small cell lung cancer (NSCLC), Hodgkin lymphoma, Merkel cell carcinoma, renal cell carcinoma (RCC), urothelial carcinoma, and head and neck cancers (Boutros et al., 2016; Topalian et al., 2012). Anti–PD-1 antibodies have also shown promise for patients with triple-negative breast cancer and metastatic colorectal cancer with mismatch repair deficiency (Easton, 2017). With positive clinical outcomes and improved understanding of the pathobiology, anti–PD-1/PD-L1 medications continue to be approved by the FDA as first-line monotherapy and combination therapy.

PD-1/PD-L1 are key immune checkpoints that are located on tumor cells and stromal cells activated by T cells, which aid in immunosuppression (Buchbinder & Desai, 2016; Topalian et al., 2012). Anti–PD-1/PD-L1 therapy (immunotherapy) works by disrupting the PD-1 and PD-L1 interactions in the tumor microenvironment, as well as in nontumor tissues (Li et al., 2017; Medina & Adams, 2016; Nishino et al., 2016a; Tada et al., 2017). Furthermore, anti–PD-1/PD-L1 can affect almost every organ in the body due to the PD-1/PD-L1 regulatory effect by the effector T cells (Diamantopoulos et al., 2017; Tada et al., 2017).

Immune checkpoint blockade is associated with unique side effects referred to as immune-related adverse events (irAEs; Naidoo et al., 2015). A severe, potentially life-threatening irAE associated with immunotherapy is pneumonitis, which may develop at any time. Pneumonitis is defined as a noninfectious inflammation to the lung parenchyma (Naidoo et al., 2015, 2017; Nishino et al., 2016b; Wu et al., 2017). Generally, irAEs with anti–PD-1/PD-L1 therapy occur less frequently compared with anti–CTLA-4 monotherapy; however, pneumonitis occurs more frequently in patients receiving anti–PD-1/PD-L1 therapy compared to CTLA-4 inhibitors (Boutros et al., 2016; Friedman, Proverbs-Singh, & Postow, 2016). A recent meta-analysis by Nishino and colleagues (2016a) evaluated 20 studies involving approximately 4,500 patients participating in PD-1 inhibitor clinical trials and found the overall incidence for pneumonitis ranged from 0% to 10.6%. Furthermore, combination therapy with other checkpoint inhibitors or therapies (such as chemotherapy and targeted therapies) with a known risk of pulmonary adverse events has been shown to increase the occurrence of pneumonitis (Boutros et al., 2016; Naidoo et al., 2015).

Nishino and colleagues (2016c) found the overall incidence of pneumonitis for patients on anti–PD-1/PD-L1 combination therapy was higher compared to monotherapy (6.6% vs. 1.6%, *p* < .001). Patients who receive radiotherapy for pulmonary lesions prior to anti–PD-1/PD-L1 treatment may also be at greater risk for treatment-related pneumonitis (Lu & Liu, 2017). Furthermore, preexisting pulmonary damage from tobacco use, radiation, idiopathic pulmonary diseases, certain pharmacotherapies (taxanes, gemcitabine, tyrosine kinase inhibitors), in addition to age and increased tumor burden, may also increase the risk of pneumonitis (Chuzi et al., 2017; Nishino et al., 2016c; Weber et al., 2015).

## CLINICAL SYMPTOMS OF PNEUMONITIS

With all anti–PD-1/PD-L1 agents, fatigue, pyrexia, chills, flu-like symptoms, and infusion reactions are typical (Eigentler et al., 2016; Naidoo et al., 2015; Weber et al., 2015). The challenge for advanced practice providers (APPs) is to distinguish expected side effects from severe adverse events, and evaluate differential diagnoses, such as pneumonitis, pneumonia, and cryptogenic organizing pneumonia (COP), all of which require different treatments.

Pneumonitis is a diagnosis based on clinical symptoms and exclusion, and is often misdiagnosed as pneumonia or other pulmonary infections (Powell, 2017). The most common initial symptoms are persistent nonproductive cough (53%), dyspnea (35%), fever (12%), and chest pain (7%; Hassel et al., 2017). Up to one-third of patients with pneumonitis are asymptomatic (Brahmer et al., 2018; Eigentler et al., 2016; Nishino et al., 2016a, 2016c). Pneumonitis can develop within days after initiation of therapy, with an earlier median time of onset for those receiving combination therapy than those receiving monotherapy (2.7 vs. 4.6 months, *p* = .02; Naidoo et al., 2017). However, research has shown that pneumonitis can occur up to 24 months after starting therapy (Naidoo et al., 2015; Nishino et al., 2016b).

Patients with immune-related pneumonitis may experience additional irAEs, including dermatitis, colitis, and endocrinopathies, such as hypophysitis and thyroiditis (Eigentler et al., 2016; Weber et al., 2015). Research by Naidoo and colleagues (2017) reported that 58% of patients (n = 43) diagnosed with pneumonitis at two institutions experienced other immune-related adverse events, with skin rash being the most common (n = 8). This is important because APPs treating patients with immunotherapy need to understand that more than one irAE can occur at the same time (Weber et al., 2015).

## DIAGNOSIS

The Common Terminology Criteria for Adverse Events (CTCAE) version 4.03 is the standard classification and severity grading scale for adverse events in cancer therapy, clinical trials, and oncology settings based on clinical symptoms and objective findings (National Cancer Institute and National Institutes of Health, 2009). It provides the framework for toxicity grading of irAE symptoms, which can then be used to follow management algorithms (Eigentler et al., 2016; Michot et al., 2016; Mistry, Forbes, & Fowler, 2017; Naidoo et al., 2015). The definition of pulmonary toxicity according to the CTCAE is as follows: Grade 1 is defined as when the patient is asymptomatic with no chest image finding, grade 2 for mild presenting symptoms that limit the patient’s activities of daily living, grade 3 for worsening or severe symptoms that limit self-care, and grade 4 for life-threateningsymptoms (National Cancer Institute and National Institutes of Health, 2009).

Chest computed tomography (CT) is preferred over a standard chest x-ray to aid in the diagnosis of pneumonitis (Nishino et al., 2016a). Radiographic findings on chest CT, in addition to clinical symptoms (i.e., new onset dyspnea, cough), aid in toxicity grading (Nishino et al., 2016a). In a retrospective study of 20 patients with anti–PD-1–induced pneumonitis, CT findings showed more extensive pneumonitis in the lower lobes compared to the middle and upper lobes (Nishino et al., 2016a). Although immunotherapy (CTLA-4, PD-1/PD-L1) has been associated with sarcoid-like pulmonary changes including lymphadenopathy, radiographic imaging can present with varied radiographic findings (Chuzi et al., 2017). Among the specific CT findings, ground-glass opacities (GGOs) were present in 13 of the 20 (65%) patients, and all patients presented with GGOs in a COP pattern (Nishino et al., 2016a).

There is some debate as to whether a diagnostic bronchoscopy is required prior to initiation of treatment to visualize inflammation and to rule out infection (Eigentler et al., 2016; Naidoo et al., 2015; Weber et al., 2015). Furthermore, there is no set standard for when to perform bronchoscopy to diagnose pneumonitis. Bronchoalveolar lavage (BAL) obtained via flexible bronchoscopy can provide information to the clinician about infectious, inflammatory, and immunologic processes at the alveolar level through analysis of the BAL fluid by cell counts, cultures, and various histochemical tests (i.e., human herpesvirus 6 [HHV-6], vesicular stomatitis virus [VSV], cytomegalovirus [CMV]; Meyer et al., 2012). Because the diagnosis of pneumonitis is one of exclusion, high-dose steroids can be used to distinguish inflammation from infection in patients who are not responding to antibiotics (Wu et al., 2017).

## CLINICAL MANAGEMENT

Depending on the toxicity grade, patients with immunotherapy-induced pneumonitis are generally treated with high-dose corticosteroids, with a median treatment time of 4 to 6 weeks (Diamantopoulos et al., 2017; Michot et al., 2016; Mistry et al., 2017; Nishino et al., 2016a). Table 1 identifies the list of authors who have published management algorithms for the diagnosis and treatment of immunotherapy toxicities, including pneumonitis. For patients with grade 1 toxicity, anti–PD-1/PD-L1 treatment is held until the chest CT findings resolve. Often, no intervention is needed until patients have grades 2 to 4 toxicity. Patients with grade 2 toxicity are considered at moderate severity and treated with oral prednisone at 1 mg/kg/day; those with higher toxicity (grades 3/4) require 2 to 4 mg/kg/day of intravenous methylpredisone (Eigentler et al., 2016; Mistry et al., 2017; Naidoo et al., 2015; Nishino et al., 2016c; Weber et al., 2015). The algorithms indicate that the treatment of patients with grade 3 (severe) or grade 4 (life-threatening) toxicity should include infectious disease and pulmonary consultations for further evaluation and bronchoscopy (Eigentler et al., 2016; Michot et al., 2016; Mistry et al., 2017; Naidoo et al., 2015). Table 2 summarizes the treatment and management of pneumonitis by grade and describes interventions needed to treat pneumonitis, when to hold or discontinue immunotherapy treatment, duration of treatment based on grade, and follow-up recommendations.

**Table 1:**
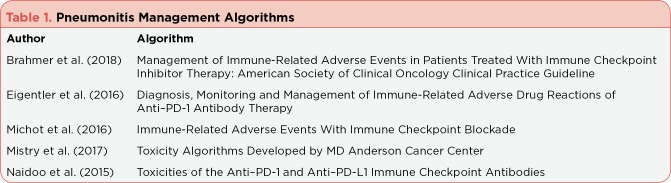
Pneumonitis Management Algorithms

**Table 2:**
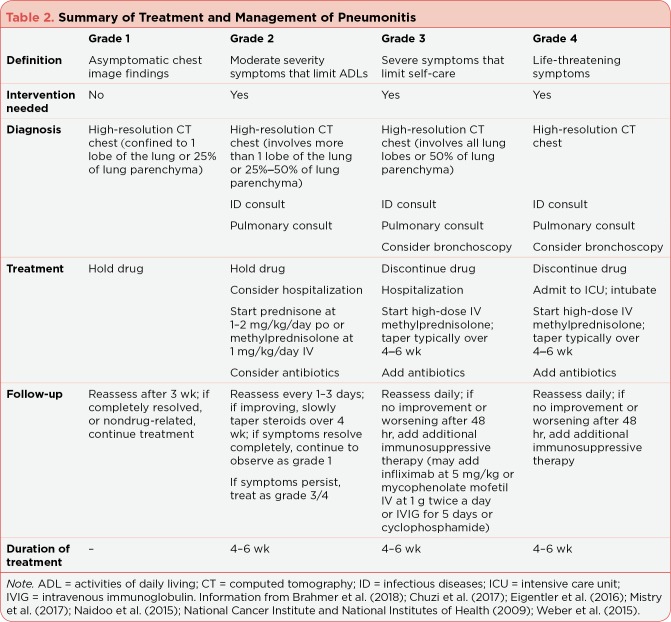
Summary of Treatment and Management of Pneumonitis

## DISCUSSION

Pneumonitis typically develops within 8 weeks after the initiation of therapy (Nishino et al., 2016c). It is important for APPs to be aware of the possibility that pneumonitis can develop any time after the initiation of therapy and to be vigilant for the presenting symptoms. The patient outlined in the case study developed pneumonitis initially at week 2, and on reinitiation of therapy, developed pneumonitis within 4 days.

As with the patient described in the case study, findings on CT imaging may be erroneously interpreted as tumor progression or infectious pneumonia. Lung events, such as pneumonitis, are often the main reason for the discontinuation of anti–PD-1/PD-L1 therapy (Michot et al., 2016). As a result, an increased awareness by radiologists and APPs is necessary to adequately diagnose pneumotoxicity related to the use of immunotherapy.

Treatment and follow-up of irAEs present a challenge in the immuno-oncology practice. Treatment algorithms for irAEs are empiric in approach and are often determined by practice settings and organizations. Some practice settings have established a consensus on the treatment of pneumonitis, leading to institutional guidelines. However, no prospective clinical trials have been identified that determine an optimal treatment approach for the management of pneumonitis and other irAEs. In February 2018, new guidelines for the management of irAEs in patients treated with immune checkpoint inhibitor therapy were published by the American Society of Clinical Oncology after a systematic review by a multidisciplinary, multiorganizational panel of experts (Brahmer et al., 2018). The recommendations for the management of pneumonitis addressed in the guidelines advise clinicians to hold immunotherapy until the patient’s pneumonitis is grade 1 or less, and permanently discontinue immune checkpoint inhibitor therapy for any patients experiencing a grade 3/4 toxicity (Brahmer et al., 2018). The National Comprehensive Cancer Network (NCCN) also provides immunotherapy teaching and monitoring tools that can be utilized by patients and providers to monitor known toxicities seen with immunotherapy (NCCN, 2017).

As novel biologic immunotherapy agents continue to emerge as the gold standard for the treatment of cancer, there is the potential for increasing rates of adverse events. Current guidelines rely on expert consensus to address irAE management; therefore, continued research on the monitoring, diagnosis, and treatment of immunotherapy toxicities is needed to strengthen the recommendations.

**Implications for Advanced Practice Providers**

The case study illustrates the difficulty in diagnosing and managing immunotherapy-induced pneumonitis. Clinicians need to be mindful of the pneumonitis risk with anti–PD-1/PD-L1 agents and factors that may increase a patient’s risk (combination therapy, solid tumors [NSCLC, RCC], smoking, age), evaluating any new symptoms as treatment related. As front-line clinicians, APPs are positioned to identify such toxicities in their patients because they often see patients at each visit and can recognize new symptoms and subtle changes. Clinical education is needed for providers caring for patients receiving immunotherapy to identify, grade, and manage the various toxicities. Although national guidelines have not been adopted, algorithms have been developed to aid in the management of these patients. In addition, NCCN has provided a robust immunotherapy teaching tool that APPs can utilize to educate patients for early detection of toxicities.
